# Can Rules in Technical-Tactical Decisions Influence on Physical and Mental Load during Soccer Training? A Pilot Study

**DOI:** 10.3390/ijerph18084313

**Published:** 2021-04-19

**Authors:** Tomás García-Calvo, Juan José Pulido, José Carlos Ponce-Bordón, Miguel Ángel López-Gajardo, Israel Teoldo Costa, Jesús Díaz-García

**Affiliations:** 1 Faculty of Sport Sciences, University of Extremadura, Av. of University S/N, 10013 Caceres, Spain; jjpulido@unex.es (J.J.P.); jponcebo@gmail.com (J.C.P.-B.); malopezgajardo@unex.es (M.Á.L.-G.); jdiaz@unex.es (J.D.-G.); 2 Centre of Research and Studies in Soccer, Universidade Federal de Vicosa, 36.571-000 Viçosa, Brazil; israel.teoldo@ufv.br

**Keywords:** monitoring, soccer constraints, small-sided games, training interventions, training load

## Abstract

This study aimed to analyze the effects of rules limitations in pass decisions during soccer tasks on physical and mental load reported by players. Participants were 40 semiprofessional Spanish soccer players (*M*_age_ = 22.40, *SD* = 2.25) from two male teams. Two training sessions with four tasks (same tasks with different score system: two maintaining ball possession games with goalkeepers, and two maintaining ball possession games) in counterbalanced order between teams were completed. To achieve a goal during limitation tasks, a minimum number of players had to participate in the passes before the goal. Internal (perceived effort and heart rate) and external physical load (distances), mental load (validated adaptation of the NASA-TXL) and fatigue (VASfatigue) were quantified. Paired t-test and magnitude-based inference were conducted. The results showed significantly higher mean speeds (*p* < 0.01), effort perception (*p* < 0.001), and mental fatigue (very likely positive) during possession games with restrictions. Additionally, performance satisfaction obtained significantly higher values with goalkeepers and pass restrictions (very likely positive). External physical load showed no significant differences between situations. The influence of mental fatigue on internal load and the complexity of the tasks could explain these results. Coaches can use this information to manipulate the training load in ecological conditions.

## 1. Introduction

Specific soccer strategies such as the number of players involved, the scoring system, or the size and orientation of the pitch could vary according to the coach’s objectives during training tasks [1]. Although these changes have usually been designed for technical-tactical objectives, this also allows the intentional manipulation of the training load by coaches in ecological conditions [2]. Thus, knowledge about the effects of these soccer strategies on training load can optimize the training process [3,4,5]. One frequently used strategy is to include constraints in pass decisions; however, no study has assessed the effect of rules in pass decisions on physical and mental load during soccer tasks. This study proposed to examine the effects of this strategy on load during soccer training tasks. 

Within the current soccer training approach, physiological, psychological, and technical-tactical elements are interrelated [6] to obtain greater specific adaptations in ecological conditions [7]. In this approach, coaches use constraints to enhance their training objectives, i.e., the maintaining ball possession games (MBPG), where teams have to achieve a certain number of consecutive passes, producing different player responses in comparison with the same tasks with goalkeepers and regular goals (MBPG-G) [8]. This is an example of how coaches could manipulate the load through the use of task constraints [9,10], such as an unbalance in the number of players for each team, specific rules in games and tasks, or modifying the field dimensions. These coaches’ modifications can differentiate task characteristics, demands, and training goals [9]. Therefore, to control the load caused by these types of tasks, it is necessary to know the specific influence of each constraint on mental and physical load [5]. This information allows a correct distribution and application of the training load [11,12].

It has been shown that larger pitch size increases the physical load of the soccer tasks in terms of total distance and heart rate [3,13,14]. Additionally, the presence of jokers decreases the physical load with higher values of walking time [15,16], whereas the presence of goalkeepers increases the distance covered during tasks [17]. However, the condition in which each player can perform a maximum number of touches on the ball decreases the walking time and distance compared to normal touch conditions [15,18]. On the contrary, to our knowledge, few studies [10,19,20,21] have analyzed the influence of these soccer-specific strategies on mental load and fatigue, although, it is demonstrated that mental fatigue decreases specific soccer performance during small-sided Games. Specifically, mental fatigue increases the number of soccer technical errors [22], impairs the spatial distribution (distances between players) [23], and decreases the physical performance and accuracy of technical-tactical decisions [4,24,25]. In this regard, it has been shown that awarding an extra point (at the beginning) or a double extra point (in the final minutes) when the points were obtained in certain time intervals produces higher levels of mental load than the habitual scores [10]. Additionally, the MBPG-G score system showed higher values in the mental load than the MBPG [19]. Furthermore, coaches’ active participation through the use of general encouragement increases mental fatigue compared to tasks where coaches adopt a passive attitude [20]. In addition, the time constrains to achieve the goal modifying the score of training matches and tasks duration also increases mental fatigue [21].

However, to our knowledge no previous studies have analyzed the influence of added rules in pass decisions (e.g., all players must touch the ball to score a valid goal) on physical and mental load, although this strategy is frequently used during soccer tasks. First, we hypothesize (1) that the restrictions in pass decisions will increase the internal and external physical load of the soccer tasks. To justify this hypothesis, we highlight that previous studies that used constraints like the limitation of ball touches or unbalance have observed an increase in one vs. one duels, the distance covered, and Rated Perceived Exertion Scale (RPE) values [15,26,27]. Additionally, in our opinion, to achieve the objectives during pass limitations tasks, players must optimize the space and increase the speed of the ball, which can increase the physical demands [27]. Secondly, we hypothesize (2) that the mental load and mental fatigue of the soccer tasks will increase with the use of the constraint in pass decisions. This assumption is based on previous studies reporting increases in the attention level [5] and cognitive demands in highly-complex environments [10] or decreases in the motivation levels [28] due to the frustration caused by worse performance. Moreover, the increases in mental fatigue (2) could have negative effects on physical performance (1) [29,30].

We consider that the present study could contribute information about how restrictions in pass decisions may affect the training load. This information can increase the control of the load changes produced by this frequently used constraint, so it can be important for coaches and practitioners. This study aimed to analyze the effects of rules in pass decisions on the physical and mental load and fatigue of soccer training tasks. 

## 2. Materials and Methods

### 2.1. Participants

Participants were 40 semi-professional soccer players from two Spanish male teams (*M*_age_ = 22.90, *SD* = 5.60) belonging to the Third Spanish League (*n =* 20) and the u-18 First Spanish League (*n* = 20). All of them participated voluntarily in the study. The two teams performed four training sessions per week (ranging between 90 to 100 min), and all players had a minimum soccer experience of 14 years. Respect to the inclusion criteria of the players, we consulted previous experts [24,25]. The criteria for players’ inclusion were: (1) players who regularly attended training sessions, and (2) players optimally accustomed to demanding training sessions, without recent injuries. According to these criteria, eight players (three players belonging to the senior team and five players belonging to the u-18 team) were excluded. 

### 2.2. Instruments

#### 2.2.1. Polar Team Pro System (Polar Electro, Finland)

To analyze mean and peak heart rate, mean and peak speed, distance/minute, and the number of sprints (accelerations over 2.8 m/s^2^), the Polar Team Pro, a global position system (GPS) was used. This technology is based on a signal concentration system of different Polar brand sensors, designed for the control and monitoring of physical activity in collective sports like soccer or basketball. This technology has been validated [31,32] and used in previous studies that registered the physical load of soccer tasks [10,19] and is currently one of the most used instruments for the quantification of the physical load in this context [33].

#### 2.2.2. Rate of Perceived Exertion

The Rated Perceived Exertion Scale (RPE) was used to quantify the perception of effort by soccer players. This instrument has been used to control the internal physical load, through the registration of the player’s exhaustion level after a physical or sports activity. The RPE includes values ranging from 0 (*not at all tired*) to 10 (*maximum exhaustion*), and its use and accuracy in soccer tasks has been proved [34].

#### 2.2.3. NASA-Task Load Index

To analyze the mental load, an adaptation of the NASA-Task Load Index (NASA-TLX) was used. The original NASA- TXL is one of the most used scales in organizational psychology. Specifically, this adaptation asked about specific soccer-related mental and physical effort, time pressure, performance satisfaction, general effort, unsafety, and interaction. This instrument includes values from 0 (*no load*) to 10 (*maximum load*) for each item described. The validity of this instrument in soccer has been demonstrated in previous studies [10,19] and it was validated [35,36]. The internal consistency (obtained by the mean value of these two times) of this scale was acceptable (Cronbach’s alpha = 0.75) with adequate temporal stability (test–retest r = 0.90).

#### 2.2.4. Visual Analog Scale (VASfatigue)

The Visual Analogue Scale 100 (VAS100) was used to quantify the mental fatigue perception of the players [37]. Originally, this procedure established a scale that includes values from 0 (*no fatigue perceived*) to 100 (*maximum fatigue perceived*) and players indicated their general fatigue perception. Participants were instructed to “Please mark the point in the line that represents your current state of mental fatigue.” The accuracy of this scale has been proven in soccer samples for the purpose described herein [38].

### 2.3. Study Design and Procedures

All research procedures were conducted in accordance with the Declaration of Helsinki (World Medical Association, 2013) and had the approval of Ethics Committee for Research with Human Beings. (approval number: 93/2020). All participants were informed about the objective of the study and signed informed consent. 

Two training sessions (A and B) with four tasks were completed by each team. This intervention was carried out at the mid-season phase (from January to March in the Spanish national competitions). The first experimental session was developed three days after the last match (on Wednesday), whereas the next match was played three days after the first experimental session had finished. The second experimental session was performed exactly one week after (also on Wednesday) the first session was performed, with the same pre- and post-match rest days, as explained. These training sessions included the same type of task with a modification in the score system constraint: Tasks 1 and 2 were MBPG, whereas Tasks 3 and 4 were MBPG-G. A restriction in pass decisions was included in one of each type of task per session (one MBGP and MBPG-G with pass limitations and one MBPG and MBPG-G without pass limitations per session). The manipulation of the limitation in pass decision was implemented by the next condition: a minimum number of players (at least three jokers, in this case) had to participate in the passes before achieving the goal (goal was not valid if this condition was not met). Additionally, to control the residual effects, the tasks were presented in counterbalanced order (see Table 1) between the teams (Team 1: Session A–B; Team 2: B–A). The inter-task rest time was two minutes between T1–T2, and T2–T3, and four minutes between T3–T4, and T4–T5. The inter-task time was used by the players to complete the VAS-100, the adaptation of the NASA-TXL, and RPE. The sample had previously experience with these instruments, as they completed an initial measure, which was not taken into account for the investigation, and this ensured that the scales were understood.

### 2.4. Data Analysis

The statistical program SPSS 25.0 (2017) and Hopkins’ (2017) specific pre-post cross-over spreadsheet were used to analyze the data obtained [39]. Data were expressed as mean and standard deviation values for all variables described. A paired t-Test was performed for each variable and pair of tasks (e.g., mental load in Task 1 of Session A and Task 2 of Session B compared with mental load in Task 2 of Session A and Task 1 of Session B). Significant levels were set at 0.1%, 1%, and 5%. Additionally, the magnitude of change in terms of effect sizes (ES) [40], was calculated with the spreadsheet named [37]. The ES were classified as: trivial (<0.2), small (0.2–0.6), moderate (0.6–1.2), large (1.2–2.0) and very large (>2.0) [41]. Magnitude-based inferences (MBI), using the confidence intervals, were used to determine the possible benefit, like positive or negative changes, of the mental and physical load and fatigue between tasks. The smallest worthwhile change (SWC) to assess the change for variables between tasks was set at an ES of 0.2 [41]. Moreover, a qualitative analysis of the changes was performed: 0.5% to 5%, very unlikely; 5% to 25%, unlikely; 25% to 75%, possibly; 75% to 95%, likely; 95% to 99.5%, very likely; and > 99.5%, most likely [42]. The use of these two types of statistical analysis (*p*-values and MBI) is based on Holgado et al.’s affirmation according to previously reported data about the statistical negative effects of mental fatigue on physical performance or the fatigue that could have been caused by statistical errors [43]. 

## 3. Results

### 3.1. Internal Physical Load

The comparison between internal physical load values is shown in Table 2. Significant differences were found in peak heart rate and RPE during MBPG between the two conditions, with higher values in MBPG tasks with the use of pass constraint. For internal physical load values, no significant differences were found for MBPG-G between normal and pass restriction conditions. These results agree with the results shown by the MBI between these two conditions.

### 3.2. External Physical Load

Table 3 presents the results of external physical load. Distance/minute and mean speed presented significant changes in the MBPG score system. In this case, distance/minute showed higher values without pass limitations, whereas the mean speed was higher in MBPG with pass limitations. No significant differences in the MBPG-G score system was observed in these variables between the two conditions. The results obtained by the MBI analysis supported the findings with unclear changes in peak speed and number of sprints for MBPG and all variables during the MBPG-G. 

### 3.3. Mental Load and Fatigue

Finally, descriptive values and the comparison of pairs of tasks for mental variables are presented in Table 4. According to the *p*-value analysis, only time pressure in the MBPG showed significant changes, with higher values in the limitation tasks. However, according to the MBI analysis, mental effort and fatigue for MBPG and performance satisfaction during MBPG-G showed very or most likely changes with higher values in tasks with pass limitations. Mental effort represents the real mental resources that the players use to achieve the goal and it produces mental fatigue Thus, the relation between these variables is clear. The rest of the mental variables (unsafety and interaction) compared for these two types of tasks did not show relevant differences (either with *p*-values or MBI analysis), although all results were higher for these variables in pass limitation conditions, both in the MBPG and the MBPG-G score system.

## 4. Discussion

The purpose of this study was to analyze the effects of limiting pass decisions on physical and mental load and fatigue during soccer training tasks through a rule stating that the goal is only valid if a minimum number of players participate in the passes before achieving the goal. Thus, we proposed two training sessions with the same tasks (with or without the constraint) and compared their results. The main findings of the study suggested that RPE and mental fatigue levels were affected by these constraints because higher values were found in these variables using the limitation, especially during the MBPG score system tasks. However, the external physical load reported by players was not affected or decreased by the use of constraints in pass decisions, either in MBPG or MBPG-G.

We expected (Hypotheses (1) that the limitation in pass decisions would increase internal and external physical values. Indeed, internal load results were higher during the limitation tasks. However, contrary to the hypothesis, most of the external physical values were higher or unchanged in no-limitation tasks. Previous research showed synergistic increases/decreases both in internal and external physical load with the use of soccer constraints like the size of the pitch [2,11,15]. However, we cannot consider these constraints as similar to pass constraints decisions. Other previous constraints and tactical behaviors more closely related to this constraint such as player’s unbalanced or the limitation in the ball touches can increase the external physical load through the players’ distributions, the increase in the speed of the ball or the type of defender marking derived from the tasks and limitations. These constraints showed increases both in internal and external load [15,26]. However, the level of the participants (semi-professionals) or the complexity of the task designed could be the key to the results of the present study, due to a greater number of errors in the pass and less effective time of practice [44]. This could explain the important decreases shown in the MBI analysis for distance/minute. 

On another hand, the disagreement between the decreases in external load and the increases in the internal load values could be explained by the mediated effect of mental fatigue. This explanation is justified by the psychobiological model. This model is characterized by increases in internal load (RPE) values without increases in external load [45]. According to this model, the increase in internal physical load (RPE) observed during these situations is mediated by the mental load increases, and according to these authors, the external physical not changed. These statements agree with the results of the present study, which showed unchanged/decreased values for external load and important increases in the internal load values. Another possible explanation is that the decreases in the distances covered with tactical objectives are caused by the effects of mental fatigue on physical performance. This behavior was observed by Coutinho et al. (2018) but, in our opinion, this explanation is improbable in the present study due to (i) the duration of the task and (ii) the team’s tactical behavior is more influenced by the objective of the task.

Concerning mental values, we expected (Hypothesis (2) that the limitations in pass decision would increase mental load and fatigue. Specifically, the results confirmed that mental and physical effort, time pressure, and mental fatigue increased in MBPG. Additionally, positive and significant changes in performance satisfaction were obtained for MBPG-G. The increases in mental load values due to the pass decision constraint could be influenced by the extra mental effort that this limitation produces in players because they must increase their attention and cognitive levels to achieve the objectives and conditions of the training tasks. Other previous studies confirmed that the task’s entropy can increase with the use of soccer constraints like the scoring system [10] or the task’s objective [19]. However, contrary to the results shown by Ponce-Bordon et al. (2020), these constraints have a higher effect in MBPG than in MBPG-G. These results could be explained by the increase in the complexity of the development or the higher level of stress caused by these types of tasks [46]. These statements coincide with the increases in performance satisfaction during MBPG-G.

Concerning mental fatigue, it is difficult to find studies to discuss the results found, because most of the studies published do not use specific soccer strategies. In this sense, Thompson et al. (2020) proved that the use of Stroop tasks before measuring sports performance has increased knowledge about mental fatigue, but mental fatigue accumulation does not occur before matches; the accumulation of mental fatigue occurs during and after soccer matches. The importance of studies that increase the information and practical applications about how coaches can manipulate mental fatigue with specific soccer strategies has been highlighted by these authors. Moreover, the control of these types of fatigue is very important because an important increase in the accumulation of mental fatigue levels can reduce soccer performance [12].

### 4.1. Study Limitations and Future Research

Probably, the main limitations of the study were the exclusion of the previous training mental fatigue levels and the effective time of practice. In future research, the quantification of prior mental fatigue levels (before the task) could increase the information obtained. Additionally, the inclusion of the effective time of practice and information about all types of fatigue. Another interesting aspect is the implementation of this intervention at different moments of the season (early, mid, or end-season), because the training load is not the same during the whole season. The different influence of this constraint on the types of tasks may also be considerable.

On the other hand, in future studies it could be interesting to analyze the implementation of this constraint during more training sessions or other types of tasks, different from MBPG and MBPG-G, comparing the types of tasks. Additionally, it would be interesting to compare the fatigue that different tasks induced. Future research should examine the differences between these results and the findings obtained with professional players or even the quantification of the effective practice time.

### 4.2. Practical Implications 

In our opinion, these results add important information for researchers, practitioners, and coaches about the use of this constraint. The use of this limitation in pass decisions is a frequently used strategy during soccer training sessions, and based on the effects reported in this research, coaches can use this specific soccer constraint to manipulate the load according to their objectives. 

## 5. Conclusions

The present study confirmed that the use of pass restrictions during soccer tasks may modify the effort perceptions and mental fatigue reported by players during soccer training sessions. Specifically, these variables increase with the use of these restrictions, with higher effects in MBPG tasks than in MBPG-G. These increases are probably mediated by the effect of mental fatigue on internal physical load, because the values of the external physical load were not modified. The cause of these unchanged values of external values could be the decreases in effective practice time, which could be caused by the complexity of this constraint or the player’s level. 

## Figures and Tables

**Table 1:** Design of the investigation. Task description and order.

Tasks	Description	Order
T1	Possession. 6 + 2 vs. 6 + 2. Field 40 × 20 m. Jokers located in lateral areas of the pitch. Jokers must not pass the ball to each other. Each 5-consecutive passes = 1 goal. 10 min long. Jokers switched every 2.5 min.	The first task in Session A, and the second task in Session B
T2	Possession. 6 + 2 vs. 6 + 2. Field 40 × 40 m. Jokers located in lateral areas of the pitch. Jokers must not pass the ball to each other. Each 5-consecutive passes = 1 goal that was only valid if at least 3 jokers participated. 10 min long. Jokers switched every 2.5 min.	The second task in Session A, and the first task in Session B
T3	Match 6 + 2 vs. 6 + 2. Field 40 × 40 m. Jokers located in lateral areas of the pitch. Match with Goalkeeper. Normal goal. 10 min long. Jokers switched every 2.5 min.	The third task in Session A, and the fourth task in Session B
T4	Match 6 + 2 vs. 6 + 2. Field 40 × 40 m. Jokers located in lateral areas of the pitch. Match with Goalkeeper. Normal goal, but only valid if at least 3 jokers participated. 10 min long. Jokers switched every 2.5 min.	The fourth task in Session A, and the third task in Session B

*Note*: T1 = Task 1, T2 = Task 2, T3 = Task 3, T4 = Task 4.

**Table 2:** Internal physical load results between pairs of tasks.

		T1–T2		T3–T4	
Variables		NOPR	PR	NOPR	PR
Mean Heart Rate	*M SD*	156.30 ± 12.43	158.13 ± 13.40	157.75 ± 12.74	158.33 ± 13.91
	t(*p*)	−1.45(0.16)		−0.30(0.76)	
	ES	0.14		0.04	
	%Change	1.17		0.32	
	%+/trivial/-QI	91/0/9 Unclear		60/0/40 Unclear	
Peak Heart Rate	*M SD*	175.63 ± 11.09	178.70 ± 11.27	179.25 ± 10.57	181.48 ± 15.62
	t(*p*)	−3.16(**)		−1.54(0.13)	
	ES	0.27		0.20	
	%Change	1.75		1.31	
	%+/trivial/- QI	100/0/0 Most Likely +ive		93/0/7 Unclear	
RPE	*M SD*	5.95 ± 1.52	6.43 ± 1.17	6.89 ± 1.21	7.18 ± 1.15
	t(*p*)	−4.69(***)		−1.09(0.28)	
	ES	0.33		0.15	
	%Change	8.07		3.34	
	%+/trivial/-	100/0/0		85/0/15	
	QI	Most Likely +ive		Unclear	

*Note*. T1 = Task 1, T2 = Task 2, T3 = Task 3, T4 = Task 4. NOPR = no pass restriction, PR = pass restriction. ES = effect size, QI = qualitative inference, +ive = positive. *** *p* < 0.001, ** *p* < 0.01.

**Table 3:** External physical load results between pairs of tasks.

		T1–T2			T3–T4	
Variables		NOPR	PR		NOPR	PR
Distance/Minute	*M SD*	104.10 ± 17.13	96.58 ± 17.06		94.05 ± 19.11	91.10 ± 17.34
	t(*p*)	3.87(***)			0.97(0.34)	
	ES	−0.44			−0.12	
	%Change	−7.22			−2.83	
	%+/trivial/-	0/0/100			23/0/77	
	QI	Most Likely–ive			Unclear	
Peak Speed	*M SD*	22.04 ± 2.04	21.93 ± 2.69		22.65 ± 2.56	23.62 ± 2.73
	t(*p*)	0.29(0.77)			−1.46(0.15)	
	ES	−0.08			0.35	
	%Change	4.04			4.28	
	%+/trivial/-	31/0/69			92/0/8	
	QI	Unclear			Unclear	
Mean Speed	*M SD*	5.95 ± 1.52	6.43 ± 1.17		5.90 ± 1.17	5.72 ± 1.06
	t(*p*)	3.56(**)			0.91(0.37)	
	ES	0.33			−0.08	
	%Change	8.07			−2.67	
	%+/trivial/-	100/0/0			23/0/77	
	QI	Most Likely +ive			Unclear	
	*M SD*	1.15 ± 1.42		1.60 ± 1.85	1.80 ± 1.77	1.88 ± 1.83
Sprints Number	t(*p*)	−2.26(*)			−22(0.82)	
	ES	−0.04			−0.27	
	%Change	39.13			−16.11	
	%+/trivial/-	45/0/55			16/0/84	
	QI	Unclear			Unclear	

*Note*. T1 = Task 1, T2 = Task 2, T3 = Task 3, T4 = Task 4. NOPR = no pass restriction, PR = pass restriction. ES = effect size, QI = qualitative inference, +ive = positive, –ive = negative. *** *p* < 0.001. ** *p* < 0.01, * *p* < 0.05.

**Table 4:** Mental load and mental fatigue results between pairs of tasks.

			T1–T2		T3–T4	
Variables			NOPR	PR	NOPR	PR
Mental Effort		*M SD*	57.50 ± 18.74	61.63 ± 17.07	62.75 ± 17.58	64.13 ± 18.15
		t(*p*)	−2.50(0.17)		−0.77(0.45)	
		ES	0.16		0.05	
		%Change	7.52		2.20	
		% (+/trivial/-) QI	96/0/4 Very Likely +ive		72/0/28 Unclear	
Time Pressure		*M SD*	56.00 ± 16.26	61.88 ± 16.20	60.25 ± 17.02	61.75 ± 18.14
		t(*p*)	−3.85(^***^)		−0.64(0.55)	
		ES	0.26		0.16	
		%Change	12.62		2.49	
		% (+/trivial/-) QI	100/0/0 Most Likely +ive		92/0/8 Unclear	
Performance Satisfaction		*M SD*	60.50 ± 15.22	62.25 ± 16.60	61.75 ± 18.21	64.50 ± 19.34
		t(*p*)	−1.22(0.23)		−1.15(0.26)	
		ES	0.04		0.21	
		%Change	1.34		4.45	
		% (+/trivial/-) QI	67/0/33 Unclear		99/0/1 Very Likely +ive	
Unsafety		*M SD*	32.63 ± 22.10	34.25 ± 22.83	38.38 ± 25.30	41.63 ± 26.15
		t(*p*)	−0.96(0.34)		−0.75(0.46)	
		ES	0.11		0.18	
		%Change	8.72		8.47	
		% (+/trivial/-) QI	94/0/6 Unclear		94/0/6 Unclear	
Interaction		*M SD*	56.75 ± 17.30	59.75 ± 18.64	57.63 ± 19.28	61.25 ± 18.70
		t(*p*)	−1.86(0.70)		−1.66(0.10)	
		ES	0.10		0.14	
		%Change	4.57		6.28	
		% (+/trivial/-) QI	94/0/6 Unclear		95/0/5 Unclear	
Fatigue		*M SD*	40.88 ± 23.59	42.88 ± 24.93	45.88 ± 24.44	46.63 ± 24.74
		t(*p*)	−1.87(0.69)		-0.83 (0.41)	
		ES	0.08		0.02	
		%Change	5.78		2.30	
		% (+/trivial/-) QI	97/0/3 Very Likely +ive		72/0/28 Unclear	

*Note*. T1 = Task 1, T2 = Task 2, T3 = Task 3, T4 = Task 4. NOPR = no pass restriction, PR = pass restriction. ES = effect size, QI = qualitative inference, +ive = positive. *** *p* < 0.001.

## Data Availability

https://osf.io/wvgxc/ (accessed on 17 April 2021).
